# Comparison of Incidence of Urinary Tract Infection in Diabetic vs Non-Diabetic and Associated Pathogens

**DOI:** 10.7759/cureus.10500

**Published:** 2020-09-17

**Authors:** Sonam Ramrakhia, Kunal Raja, Kapeel Dev, Ajay Kumar, Vinesh Kumar, Besham Kumar

**Affiliations:** 1 Medicine, Liaquat University of Medical and Health Sciences, Sukkur, PAK; 2 Medicine, Mustafai Trust Central Hospital, Sukkur, PAK; 3 Internal Medicine, Shaheed Mohtarma Benazir Bhutto Medical University, Larkana, PAK; 4 Internal Medicine, Ghulam Muhammad Mahar Medical College, Sukkur, PAK; 5 Internal Medicine, Jinnah Sindh Medical University, Karachi, PAK; 6 Internal Medicine, Ghulam Mohammad Mahar Medical College, Sukkur, PAK; 7 Internal Medicine, Jinnah Postgraduate Medical Centre, Karachi, PAK

**Keywords:** diabetes mellitus, urinary tract infection, pakistan

## Abstract

Introduction

Urinary tract infections (UTIs) are common in low socioeconomic country like Pakistan. There are various factors responsible for UTI, one major factor being diabetes. This study aims to compare diabetic and non-diabetic patients, for gender association, symptoms, and organisms, with UTI.

Methods

This cross-sectional study was conducted in the medicine ward of tertiary care hospital in Pakistan from January 2019 to December 2019. For urine analysis, freshly voided 5-10 ml of clean midstream urine specimens was collected in a sterile container. Samples were sent to the lab immediately, A colony count of ≥10^5 ^CFU/ml was considered for the diagnosis of UTI. Culture was done if UTI was diagnosed.

Results

The overall incidence of UTI in participants of the diabetic group was significantly higher than those in the non-diabetic group (13.67% vs 6.40%; P=0.004). *Escherichia coli *was the most common organism in both the diabetic and non-diabetic groups (60% vs 72%; P=0.73). Frequency of *Klebsiella *was considerably higher in the participants of diabetes but it was not significant (23.3% vs 11.1%; P=0.29).

Conclusion

UTI was significantly higher in the diabetic population compared to the non-diabetic population. Since diabetes is prevalent in Pakistan, care of diabetes should include reducing the risk factors for UTI.

## Introduction

Urinary tract infection (UTI) is common and is usually caused by bacteria [1]. There are many causes of UTI, of which diabetes is one of the potential reasons of UTI [2], which occurs due to alteration in the immunity of diabetic patients like granulocyte dysfunction [3]. Studies have suggested that the pathogens cause UTI to adhere to the uroepithelial cells resulting in impaired intracellular calcium metabolism [4,5].

Patients may or may not elicit signs and symptoms depending upon the infection severity [6]. Patients suffering from UTI complain of nausea, vomiting, fatigue, spiking fever, chills, dysuria, urinary frequency, urgency, lower abdominal/flank/back pain, and, in severe cases, altered levels of consciousness [7]. UTI can be classified based on its location (upper and lower) and severity (uncomplicated and complicated). Uncomplicated UTI is relatively mild, occurs more commonly in females, and does not necessitate treatment through antibiotic therapy [8]. Complicated UTI has relatively severe signs and symptoms and is not easy to treat [9]. Recurrence of UTI is common either through the same microorganism's involvement, which causes relapse, or re-infection by a different microorganism [10-12]. Common pathogens involved are *Escherichia coli*, *Klebsiella *spp., *Mycoplasma *spp., *Enterobacter* spp., *Staphylococcus aureus*, *Candida albicans*, and worms like threadworms, flukeworms, etc. Of these, *E. coli *is the most common cause of UTI [13,14].

Various factors increase the risk of UTI, including diabetes mellitus. UTI in patients with diabetes is more severe and has more complication. Various factors contribute to an increase in frequency of UTI in diabetes, including weak immune system, neuropathy, and poor metabolic control [15].

To our knowledge, there is no study available that analyzes the difference in the treatment of UTI among diabetic and non-diabetic patients. Therefore, through this study, we plan to compare the pathogens causing UTI in diabetic and non-diabetic patients, which will help physicians modify their treatment strategies for UTI depending upon the presence or absence of comorbidity like diabetes in the future.

## Materials and methods

This cross-sectional study was conducted in the medicine ward of tertiary care hospital in Pakistan from January 2019 to December 2019. A total of 1,074 participants were included in the study, including 512 diabetic patients and 562 non-diabetic caregivers of diabetic patients. Participants who took any antibiotics for the last 14 days during data collection were excluded. Patients' demographics, symptoms, and diabetes status were recorded in a self-structured questionnaire.

For urine analysis, freshly voided 5-10 ml of clean midstream urine specimens was collected in a sterile container. Samples were sent to the lab immediately, where they were analyzed. A colony count of ≥10^5^ CFU/ml was considered for the diagnosis of UTI [16]. Urine of participants with colony count of ≥10^5 ^CFU/ml was sent for urine culture for identification of organism.

The analysis of data was done using the SPSS statistical software package version 23 (IBM Corp., Armonk, NY). Numerical data such as age were presented as mean and standard deviation. Percentage and frequency were used to present symptoms, gender, diabetic status, and organism. The chi-square test was used to compare the two groups. A p-value of less than 0.05 meant that there is a difference between the two groups, and the null hypothesis is void.

## Results

A total of 1,074 participants were included, who were randomized into two groups: diabetic (n=512) and non-diabetic group (n=562 (Table 1). 

**Table 1:** Patients' Demographics HbA1c, hemoglobin A1c

Demographics	Diabetes (n=512)	Non-Diabetes (n=562)
Gender: male, female	224 (43.75%), 288 (56.25%)	250 (44.48%), 312 (55.51%)
Age	46±11	51±13
HbA1c	7.08±2.24	4.91±1.06

The overall incidence of UTI in participants of the diabetic group was significantly higher than those in the non-diabetic group (13.67% vs 6.40%; P=0.004). Females had higher proportion of UTI incidence compared to males (10.6% vs 8.8%); however, when stratified for diabetes status, the result was not significant (Table 2).

**Table 2:** Incidence of Urinary Tract Infection (UTI) * means significant result, ** means non-significant result

Incidence of UTI	Diabetes	Non-Diabetes	P-value
Total	70 (13.67%)	36 (6.40%)	0.004*
Males	26 (11.60%)	16 (6.40%)	0.52**
Females	44 (15.27%)	20 (6.41%)	

Fever and dysuria were significantly higher in patients with diabetes compared to non-diabetic patients. There was no significant difference in any symptom (Table 3).

**Table 3:** Symptoms of Urinary Tract Infection * means significant result, ** means non-significant result

Symptoms	Diabetes (n=70)	Non-Diabetes (n=36)	P-value
Fever	46 (65.7%)	12 (61.11%)	0.02*
Dysuria	34 (48.55%)	6 (50.0%)	0.023*
Increased frequency	18 (25.71%)	10 (27.77%)	0.86**
Abdominal pain	16 (22.85%)	10 (27.77%)	0.93**
Vomiting	6 (8.57%)	4 (11.1%)	0.92**
Hematuria	4 (5.71%)	2 (5.55%)	0.87**
Pyuria	8 (11.42%)	4 (11.11%)	0.82**
Incontinence	18 (25.71%)	10 (27.77%)	0.86**
Retention	16 (22.85%)	4 (11.11%)	0.30**

*E. coli *was the most common organism in both diabetic and non-diabetic groups. The frequency of *Klebisella* was considerably higher in the participants of diabetes but it was not significant (Table 4).

**Table 4:** Organisms Identified in Culture NA, not applicable ** means non-significant result

Organisms	Diabetes (n=70)	Non-Diabetes (n=36)	P-value
Escherichia coli	36 (60.0%)	26 (72%)	0.73**
Klebisella	14 (23.3%)	4 (11.1%)	0.29**
Enterococcus	2 (3.3%)	0	0.54**
Pseudomonas	2 (3.3%	4 (11.1%)	0.28**
Coagulase-positive Staphylococcus	2 (3.3%)	0	0.54**
Others	4 (6.6%)	2 (5.5%)	0.87**

## Discussion

Diabetic patients are more prone to develop UTI as compared to non-diabetic patients. There are many mechanisms that may explain the higher prevalence of UTI in diabetic patients. Studies suggest that high glucose levels in urine aid in the growth of uropathogens [17]. Complications such as emphysematous pyelonephritis develop due to higher glucose levels in renal parenchyma, creating a favorable environment for bacterial colonization [18]. Higher glucose levels may disturb humoral, innate, and cellular immunity. They may also cause bladder dysfunction due to autonomic neuropathy, leading to urinary retention and stasis [19,20].

Our results show that UTI is more prevalent in females than in males due to short urethra and its proximity to anal canal [21]. Family history, i.e. UTI in first-degree relatives, plays a role in the development of recurrent UTI and pyelonephritis in females, which can be explained by genetic factors involving immunological response against uropathogens. Change in normal flora of vagina that decreased pH levels of the vagina may also contribute to UTI [22]. 

In our study, when we compared the symptoms of UTI between diabetic and non-diabetic patients, we observed that diabetic patients had relatively higher fever and dysuria, while other symptoms were similar in both the groups. This is inconsistent with Garg study in 2015, according to which fever, dysuria, pain abdomen, vomiting, hematuria, storage, and voiding were significantly higher in the diabetic group compared to the non-diabetic group [23].

In this study, the most common organism that causes UTI is *E. coli*, both in diabetic and non-diabetic participants. This organism adheres to urothelial cells by anchoring to glycolipid present on their cell membrane through P fimbriae [24]. P fimbriae is composed of many subunits, the most essential of these is FimH as it helps in invading the urothelial cells and adhesion to glycoprotein containing mannose compounds [25].

The risk of developing UTI in diabetic and in non-diabetic groups is different because of many reasons, a few of which have been listed above, which is why it is necessary to recognize these risk factors. Identifying these risk factors will help us prevent complications related to UTI in patients with diabetes.

## Conclusions

In our study, UTI was significantly higher in patients with diabetes and in female patients. The clinical symptoms of dysuria and pain abdomen were more significant in diabetic females. *E. coli *was the most common isolate in both groups, followed by *Klebsiella*. A thorough watch and aggressive outpatient management of a diabetic patient who presents with simple UTI can prevent progression to more dreaded complications and associated morbidity and mortality.
